# Characterization of the ZDSD Rat: A Translational Model for the Study of Metabolic Syndrome and Type 2 Diabetes

**DOI:** 10.1155/2015/487816

**Published:** 2015-04-16

**Authors:** Richard G. Peterson, Charles V. Jackson, Karen Zimmerman, Willem de Winter, Norman Huebert, Michael K. Hansen

**Affiliations:** ^1^PreClinOmics, Inc., 7918 Zionsville Road, Indianapolis, IN 46268, USA; ^2^Janssen Research & Development, A Division of Janssen Pharmaceutica NV, Turnhoutseweg 30, 2340 Beerse, Belgium; ^3^Janssen Research & Development, LLC, Spring House, PA 19477, USA

## Abstract

Metabolic syndrome and T2D produce significant health and economic issues. Many available animal models have monogenic leptin pathway mutations that are absent in the human population. Development of the ZDSD rat model was undertaken to produce a model that expresses polygenic obesity and diabetes with an intact leptin pathway. A lean ZDF rat with the propensity for beta-cell failure was crossed with a polygenetically obese Crl:CD (SD) rat. Offspring were selectively inbred for obesity and diabetes for >30 generations. In the current study, ZDSD rats were followed for 6 months; routine clinical metabolic endpoints were included throughout the study. In the prediabetic metabolic syndrome phase, ZDSD rats exhibited obesity with increased body fat, hyperglycemia, insulin resistance, dyslipidemia, glucose intolerance, and elevated HbA1c. As disease progressed to overt diabetes, ZDSD rats demonstrated elevated glucose levels, abnormal oral glucose tolerance, increases in HbA1c levels, reductions in body weight, increased insulin resistance with decreasing insulin levels, and dyslipidemia. The ZDSD rat develops prediabetic metabolic syndrome and T2D in a manner that mirrors the development of metabolic syndrome and T2D in humans. ZDSD rats will provide a novel, translational animal model for the study of human metabolic diseases and for the development of new therapies.

## 1. Introduction

Metabolic syndrome affects a significant proportion of the population and is becoming increasingly more prevalent in adolescents [1–4]. The syndrome embodies many components including central obesity, insulin resistance, dyslipidemia, and hypertension [3, 5]. In addition, the syndrome features a chronic low grade inflammatory state, vascular endothelial dysfunction, and a prothrombotic environment [3]. Long standing metabolic syndrome predisposes an individual to type 2 diabetes (T2D), atherosclerosis, microvasculature disease (of retina), stroke, renal injury, and neuropathy [5]. Due to the complicated mechanisms involved in the syndrome and its sequelae, current clinical standard of care embodies polypharmacological therapeutics aimed at controlling atherogenic dyslipidemia, hyperglycemia, and hypertension as well as intervening in secondary conditions such as renal dysfunction, stroke, and microvascular disease related to retinopathy. Development of new chemical entities with the potential to control more than one risk factor is hampered by the paucity of highly translational animal models.

The most frequently used rat and mouse models for obesity, metabolic syndrome, and T2D have defects in the leptin pathway. The Zucker diabetic fatty (ZDF) has been a gold standard for this disease complex and as such has many of the characteristics of the human condition but it becomes obese due a leptin receptor defect and becomes diabetic before the animals are mature. To circumvent these ZDF complications, the ZDSD/Pco (ZDSD) rat has been developed to be a more translational model of obesity, metabolic syndrome, and diabetes and to aid the study of these conditions and the development of drugs that could assist in control and treatment. The model was developed by crossing a homozygous lean Zucker diabetic fatty (ZDF) male rat with a substrain of the Crl:CD (SD) rats that had been selectively bred for high fat diet induced obesity [6, 7]. The standard Crl:CD (SD) rat is a substrain of SD rats that is significantly heavier and more obese than other lines of SD rats; a percentage of these rats is very susceptible to develop obesity, fed high fat diets [6, 7]. The original design was to combine the defect in *β*-cell gene transcription that is found in lean and obese ZDF rats [8] with the obesity of the Crl:CD (SD) model to produce an obese diabetic model that preserves the critical leptin pathway. The animals were fed regular rodent chow (Purina 5008) during the 12 years of the model development process. The offspring from the initial crosses were screened and selected for obesity, the propensity to become diabetic and the expression of the other characteristics of metabolic syndrome. This model has been selectively inbred for >30 generations.

In the current study, ZDSD rats were followed for 6 months; clinically relevant metabolic endpoints were included throughout the study to extensively characterize the phenotype and development of metabolic disease. The authors believe that the ZDSD rat displays a phenotype with close similarity to metabolic syndrome/T2D in humans further representing a “one rat, many models” tool that will enable the study of early metabolic dysregulation, overt diabetes, and late stage complications of T2D in one rat. It is the sum of the presence of all the characteristics of the human prediabetic state, T2D, and the growing number of associated comorbidities that creates the belief that the ZDSD rat is highly translational to the human condition. It is our belief that this model will reduce development and clinical costs and facilitate discovery of new agents with potential to impact multiple components of the disease. This study was designed to follow a cohort of male ZDSD rats from early in life and as they progress through obesity/metabolic syndrome into full T2D with beta cell failure. The study objectives included comparison of the model's disease progression to the development of diabetes in the human condition. This paper will be followed by a publication that will use these pieces of data to compare this progression in the ZDSD rat to what occurs in the human condition using a model similar to a published paper [9].

## 2. Research Design and Methods

### 2.1. Animals

Twenty-four male ZDSD rats (ZDSD/Pco) of comparable body weight were sourced at 6 weeks of age for the study (PreClinOmics Inc., Indianapolis, IN, USA). Animals were housed 2 per cage, fed Purina 5008 chow, and given water* ad libitum* for the duration of the study unless otherwise noted.

### 2.2. Definitions of Diabetic State

The definition of the permanent onset of diabetes in this model is quite simple, two subsequent weekly morning glucose readings of over 250 mg/dL. When this occurs, the animals will consistently continue to remain hyperglycemic and continue to get more overtly diabetic. For the purposes of this publication, three metabolic states are defined and used to describe the progressive disease states of the model that are typically used in describing the human conditions: (1) metabolic syndrome/prediabetic hyperglycemia: fed/fasted glucose levels above 125 mg/dL, 50% increase in glucose AUC in the OGTT and a 25% increase in HbA1c; (2) diabetic: fed glucose levels above 250 mg/dL, >100% increase in HbA1c and a >200% increase in AUC in the OGTT; and (3) overt diabetes: equaling or exceeding values in (2) above decreases in insulin levels and weight loss. Depending on the methodology used for glucose measurements in an experiment, the actual glucose levels may vary.

### 2.3. Assays

Body weight was recorded and whole blood was collected every 2 weeks at 6 a.m. for purposes of obtaining fed analyte values. The whole blood (nonfasted samples) was collected by tail clip at 6 a.m. between 7–31 weeks of age for assessment of fed glucose levels using StatStrips (StatStrip Xpress Glucometer, Nova Biomedical, Waltham, MA, USA). Animals were then fasted for 6 hours and a sample of lithium heparin anticoagulated blood (100–150 *μ*L) was taken from a second tail clip. HbA1c was measured in 20 *μ*L of whole blood prior to processing to plasma (AU480; Beckman Coulter, Brea, CA, USA). Glucose, triglycerides, and cholesterol were assayed on fresh plasma samples using a clinical chemistry analyzer (AU480; Beckman Coulter, Brea, CA, USA). Oral glucose tolerance tests (OGTTs) were performed monthly after a 6-hour fast using a glucose load (2 g/kg, p.o.) and sampling from the tail at 0 (prior to glucose administration), 15, 30, 60, and 120 minutes postglucose load. The 7-week OGTT was done using blood glucose (StatStrip) methodology; plasma insulin levels were assayed at each time point of the OGTT. For subsequent OGTTs, glucose was determined from fresh plasma on the AU480 and insulin levels were determined from frozen plasma using a rat/mouse Meso Scale Discovery kit (K152BZC, Rockville, MD, USA). To insure that fasting did not negatively affect the phenotype, the fasts were limited to 6 hours (6:00 a.m.–12:00 p.m.) every two weeks.

### 2.4. Statistical Analysis

Data are represented as mean ± SEM. All data analysis was accomplished using JMP Statistical Software (SAS Institute). The effect of age on measured parameters (body weight, triglycerides, Cholesterol, HbA1c, NEFA, glucose, glucose AUC, insulin, and insulin AUC) was determined using one-way ANOVA with repeated measures (*P* < 0.05) and when necessary Tukey's multirange *t*-test was performed (*P* < 0.05). The effect of age on the 5-point oral glucose tolerance tests (OGTT) was determined using two-way ANOVA with repeated measures (*P* < 0.05). A Mahalanobis distance plot and *T* ^2^ statistic were used to identify outliers based on average fed glucose levels over the course of the study. Based on this analysis, one animal was retrospectively eliminated from analysis. A technical issue regarding the collection and processing of fasted blood samples was noted at the 17-week time point. This necessitated elimination of these samples (glucose, triglycerides, cholesterol, and insulin) from analysis. Fasting plasma glucose and insulin levels were used to calculate the homeostasis model assessment of *β*-cell function (HOMA-*β*) and insulin sensitivity index (ISI). HOMA-*β* was calculated as [20 × fasting insulin (*μ*U/mL)/fasting glucose (mmol/L) − 3.5]. Insulin sensitivity was calculated from the OGTT using Matsuda index (1,000,000/square root fasting glucose × fasting insulin × glucose AUC × insulin AUC). Homeostatic assessment of insulin resistance (HOMA IR) was calculated using fasting glucose (mmol/L) × fasting insulin (*μ*U/mL)/22.5 [10, 11].

## 3. Results

### 3.1. Body Weight

**Figure 1:**
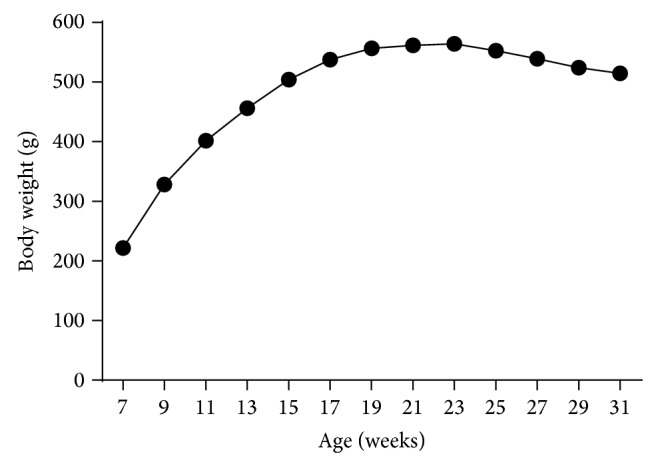
Body weight recorded biweekly throughout the study duration of 7- to 31-week-old ZDSD rats (mean ± SEM, *n* = 23, error bars included too small to see).

Male ZDSD rats weighed on average 221.9 ± 2.0 g at 7 weeks of age as study observations began. Body weights reached a plateau and peaked at 23 weeks of age (564.4 ± 8.2 g). Thereafter there was a significant (*P* < 0.0001) decline (8.2% from the peak) in body weight over the next 8 weeks (Figure 1).

### 3.2. Glucose

**Figure 2:**
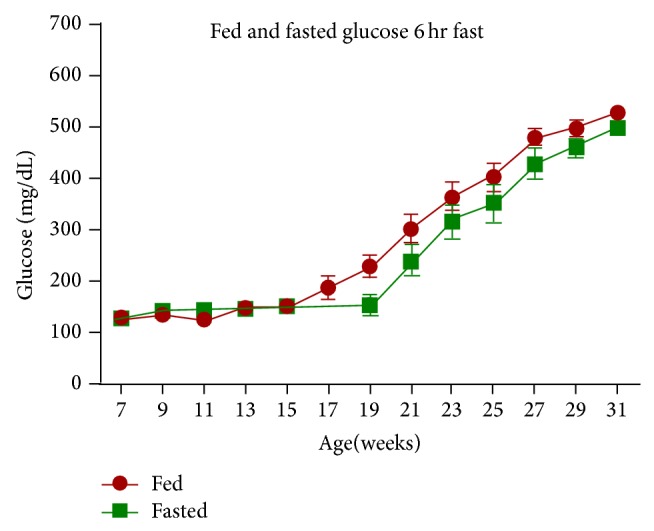
Glucose levels were taken biweekly in fed (●) (BG) or fasted (■) plasma glucose ZDSD rats throughout the study duration of rats in ages 7 to 31 weeks (mean ± SEM, *n* = 23).

Blood glucose (BG) values in the fed state were obtained from whole blood using StatStrips. BG in male ZDSD rats progressed significantly (*P* < 0.0001) from prediabetic hyperglycemia to diabetic levels with age (Table 1). Most animals (56.5%) were hyperglycemic as early as 7 weeks of age with a mean fed BG of 127.4 ± 2.3 mg/dL. BG levels rose steadily, reaching 227.3 ± 21.7 and 299.91 ± 26.4 mg/dL at 19 and 21 weeks, respectively, such that most animals (56.5%) could be classified as diabetic by 21 weeks. BG levels continued to increase such that 100% of animals were diabetic by 27 weeks of age (478.4 ± 15.6 mg/dL). BG levels rose to 527.8 ± 11.0 mg/dL at 31 weeks of age. Similarly, mild hyperglycemia in the fasted state was also observed in animals as young as 7 weeks of age (125.7 ± 2.1 mg/dL). Fasted plasma glucose levels remained relatively stable up to 19 weeks of age. Then significant (*P* < 0.0036) increases were noted (151.65 ± 15.4 versus 236.8 ± 27.6 mg/dL at 19 and 21 weeks, resp.). Fasted plasma glucose values progressed steadily to 498.1 ± 10.0 mg/dL in 31-week-old animals (Figure 2). Since fed blood glucose (BG) and fasted plasma glucose were taken using different methods, statistical comparison was not appropriate. Comparison of these two methods of glucose determination demonstrated that the BG levels run about 88% of the plasma glucose levels. This would effectively make the difference between fed BG and fasted plasma glucose significantly greater than what is shown in Figures 2 and 11. All animals that were analyzed in this study were overtly diabetic at the end of the study (as defined in methods above).

### 3.3. Glycated Hemoglobin

**Figure 3:**
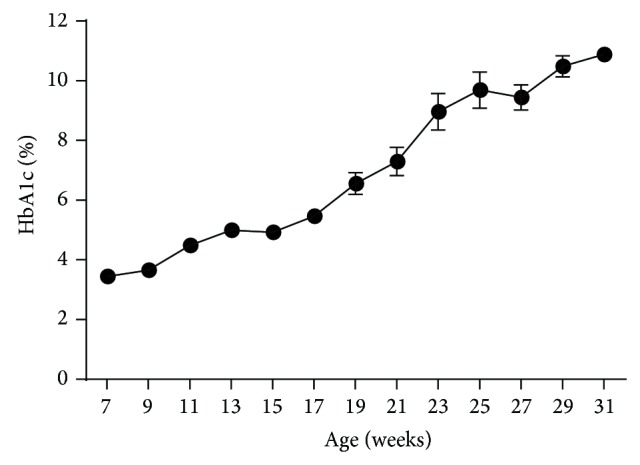
HbA1c levels in male ZDSD rats throughout the study duration of rats in ages from 7 to 31 weeks (mean ± SEM, *n* = 23).

Chronic progressive hyperglycemia with an average fed BG level of 281.5 ± 11.1 mg/dL was confirmed as evidenced by a significant (*P* < 0.0001) increase in glycated hemoglobin (HbA1c) levels taken throughout the study period. HbA1c values in 7-week-old CRL:CD (SD) control rats ranged from 3.35 to 3.60 and remained relatively stable throughout their life span (data not shown). HbA1c levels in 7-week-old male ZDSD (3.45 ± 0.03) rats were the same as those of the control animals (3.44 ± 0.03%). Despite relatively steady morning glucose levels between 7 and 15 weeks of age (Figure 2), the HbA1c levels increased by 42% from baseline values during this period (3.45 ± 0.03 versus 4.92 ± 0.05%) suggesting that there likely were unseen hyperglycemic excursions during this period (see discussion on pm glucose levels). Thereafter, HbA1c levels rose rapidly, reaching 10.88 ± 0.23% (Figure 3).

### 3.4. Glucose Levels during Oral Glucose Tolerance Tests

**Figure 4:**
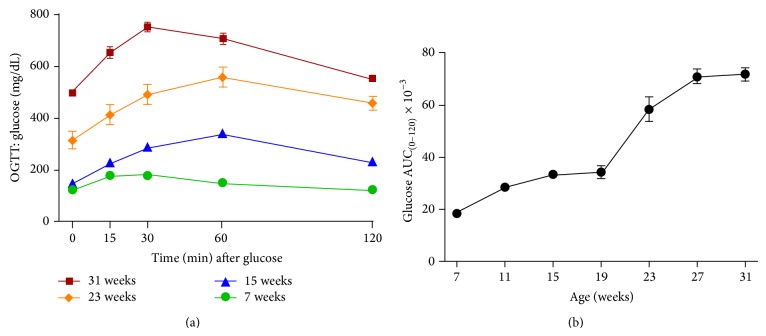
(a) Glucose levels from oral glucose tolerance tests in male ZDSD rats at 7 (●), 15 (▲), 23 (*◆*), and 31 (■) weeks of age and (b) glucose AUC calculations for all OGTTs (mean ± SEM, *n* = 23).

Oral glucose tolerance tests (OGTTs) were performed every four weeks during the experiment. For clarity purposes, only data at eight-week intervals are presented (Figure 4(a)). Significant abnormalities in glucose disposal were observed in aging ZDSD rats during performance of oral glucose tolerance tests. Glucose values taken just prior to glucose load (time zero) increased significantly (*P* < 0.0001) over time (124.9 ± 2.1, 149.4 ± 2.4, 314.7 ± 34.0, and 498.1 ± 10.0 mg/dL for 7, 15, 23, and 31 weeks, resp.). Similarly, the peak glucose excursion and the glucose levels 2 hours after administration of glucose load rose dramatically as animals aged, creating a significant (*P* < 0.0001) age-dependent increase in the area under the curve (×10^−3^ for glucose disposal, 18.4 ± 0.2, 33.5 ± 0.4, 58.5 ± 4.2, and 72.1 ± 2.4 for 7, 15, 23, and 31 weeks, resp.) (Figures 4(a) and 4(b)).

### 3.5. Insulin Levels during Oral Glucose Tolerance Tests

**Figure 5:**
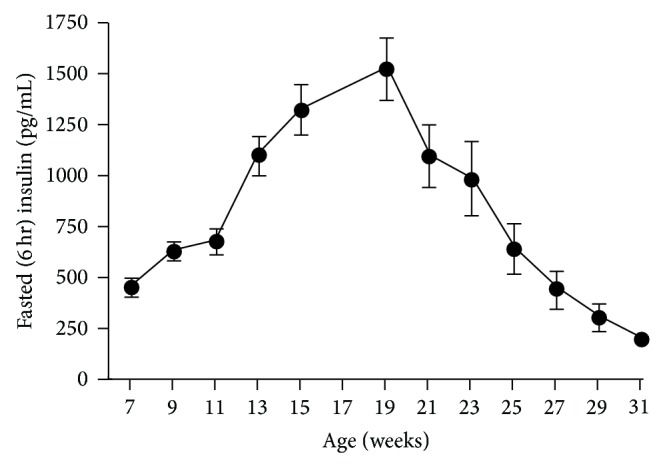
Fasted insulin levels in male ZDSD rats throughout the study duration of rats in ages from 7 to 31 weeks (mean ± SEM, *n* = 23).

**Figure 6:**
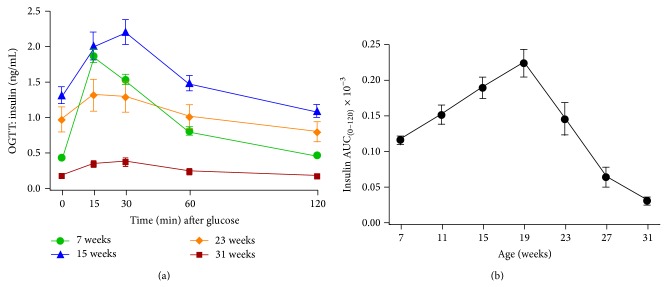
(a) Insulin levels from oral glucose tolerance tests in male ZDSD rats at 7 (●), 15 (▲), 23 (*◆*), and 31 (■) weeks of age and (b) insulin AUC for all OGTTs (mean ± SEM, *n* = 23).

In contrast to glucose values obtained during OGTT, fasted insulin values obtained just prior to glucose load increased significantly (*P* < 0.0001) in animals 7 to 19 weeks of age, after which a precipitous drop in insulin level was noted. Insulin levels in 7-week-old animals were 446.44 ± 38.9 pg/mL. Levels peaked at 19 weeks (1529.3 ± 153.3 pg/mL) and then fell significantly (*P* < 0.0001) from the peak to 189.4 ± 27.4 pg/mL in 31-week-old animals (Figure 5). In 7-week-old animals, the insulin response to glucose loading was rapid, occurring at 15 minutes, and represents a dramatic 4.3-fold increase over values prior to glucose loading (448.8 ± 37.4 versus 1935.3 ± 106.4 pg/mL). Further aging resulted in reduction of the magnitude of the insulin response in all age groups (1.7-, 1.35-, and 1.9-fold for 15, 23, and 31-week-old animals, resp.). The curve for the insulin response to glucose load revealed an increasingly flat profile as animals aged, such that insulin responses in 31-week-old animals were minimal throughout the 2-hour period. The area under the curve (×10^−3^) for the insulin response increased significantly (*P* < 0.0001) and peaked at 19 weeks followed by a significant drop from peak in 23- (*P* < 0.0016) and 31- (*P* < 0.0001) week-old animals (118.4 ± 5.3, 225.3 ± 17.5, 146.5 ± 21.7, and 32.1 ± 5.2 at 7, 19, 23, and 31 weeks, resp.) (Figures 6(a) and 6(b)).

### 3.6. Beta-Cell Function

**Figure 7:**
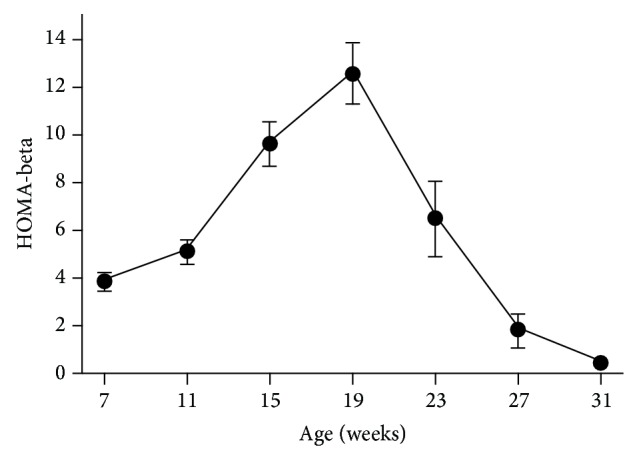
Calculated HOMA-*β* in male ZDSD rats throughout the study duration of rats in ages from 7 to 31 weeks (mean ± SEM, *n* = 23).

Fasting plasma glucose and insulin levels obtained from OGTT's were used to calculate the homeostasis model assessment of *β*-cell secretory capacity. The values followed the same time course as did the fasted insulin levels presented in Figure 3, that is, a progressive increase 7–19 weeks of age, followed by a precipitous decline to values indicating very little insulin secretory capacity at 31 weeks. HOMA-*β* values were 3.92 ± 0.31 at 7 weeks of age, peaked at 19 weeks (12.6 ± 1.22), and fell abruptly (*P* < 0.0001) to 0.44 ± 0.1 at 31 weeks, indicating only minimal secretion at 31 weeks (Figure 7).

### 3.7. Insulin Sensitivity Index (ISI)

**Figure 8:**
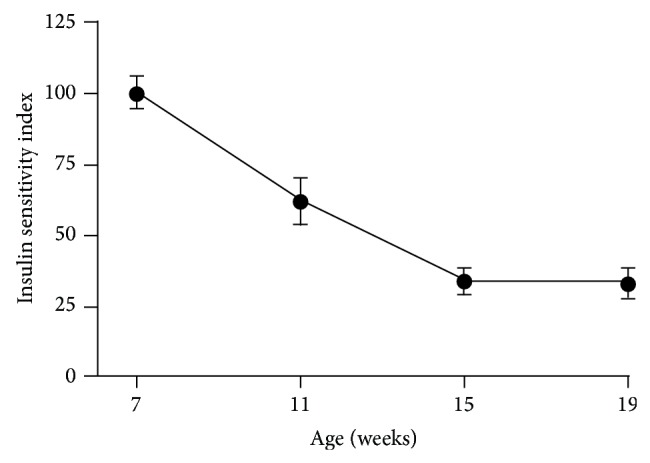
Calculated ISI in male ZDSD rats throughout the study duration of rats in ages from 7 to 19 weeks (mean ± SEM, *n* = 23).

Insulin sensitivity in ZDSD animals reflects the progressive hyperglycemia and augmented insulin secretion before beta cell loss as animals age. ISI in 7-week-old animals (99.3 ± 5.8) decreased quickly to 61.9 ± 7.5 at 11 weeks (*P* < 0.0002) and plateaued at 19 weeks (33.01 ± 4.7). This decline represents a significant 67% decrease in sensitivity within the 7–19 week old animals (*P* < 0.0001) (Figure 8).

### 3.8. Lipid Levels

**Figure 9:**
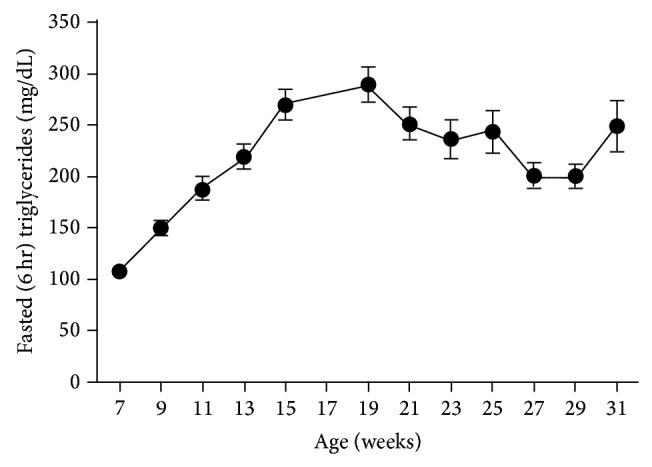
Fasted triglycerides in male ZDSD rats throughout the study duration of rats in ages from 7 to 31 weeks (mean ± SEM, *n* = 23).

**Figure 10:**
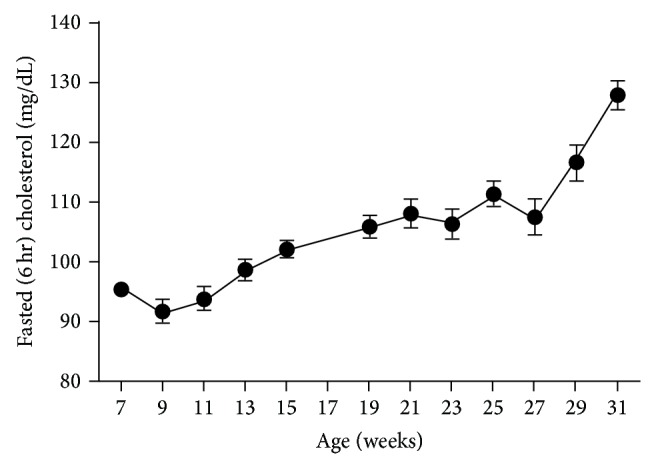
Fasted cholesterol in male ZDSD rats throughout the study duration of rats in ages from 7 to 31 weeks (mean ± SEM, *n* = 23).

Fasted triglyceride levels increased significantly (*P* < 0.0001) compared to that of 7-week-old rats and peaked at 19 weeks of age (107.5 ± 3.7 and 292.5 ± 17.8 mg/dL, resp.). A slight but not significant decrease (*P* < 0.1170) to 251.1 ± 16.7 mg/dL was apparent at 21 weeks; however, levels remained relatively stable for the remainder of the study. Average triglyceride levels were 230.3 ± 8.8 mg/dL over the last 12 weeks (Figure 9). Fasted cholesterol levels increased significantly (*P* < 0.0001) over the course of data collection, ranging from 95.4 ± 1.1 to 127.9 ± 2.4 mg/dL at 7 and 31 weeks, respectively (Figure 10).

## 4. Discussion

Over the last decade, there has been a global increase in obesity and type 2 diabetes (T2D). In 2011, there was an estimated 375 million people worldwide with diabetes [12]. Ninety percent of individuals with diabetes were reported as having T2D, largely the result of obesity and lack of physical activity (WHO, 1999, 2003). T2D is thought to occur, in large part, as a consequence of the development of metabolic syndrome [13] which presents as a cluster of conditions defined by obesity, insulin resistance, hypertension, hyperlipidemia, and hyperglycemia [14]. In humans, the natural course of T2D progresses from insulin resistance to compensatory hyperinsulinemia resulting in beta cell failure and overt diabetes [15]. Development can be initiated in humans by genetic and/or environmental factors and is thus considered a multifactorial disease [16]. Genetic polymorphisms linked to T2D have been identified in at least 3 dozen genes, most of which influence both hepatic and peripheral insulin resistance, and adipogenesis as well as beta cell mass and function [17]; however, no mutations in leptin signaling have been identified in human T2D [18, 19]. Since obesity is a major environmental factor predisposing humans to T2D [20], it becomes necessary for a translational animal model to become obese by a polygenetic mechanism.

The ZDSD rat was developed to address the disparity in disease development from the human condition that is apparent in commonly used rodent models. Previous models (Zucker fatty rat, ZDF rat,* ob/ob* mice, and* db/db* mice) have proven useful in drug development and in research in the areas of obesity, metabolic syndrome, and diabetes. However, their mutations compromise the leptin pathway [21] which is essential for the normal function of CNS obesity and feeding behavior [22]. Since leptin pathway mutations are a very rare condition in the human population, the continued exclusive use of these models will impede the studies of CNS mechanisms that are central to combating the obesity conditions in the human population. The highly translational ZDSD model satisfies this important need.

The ZDSD rat model is similar to an animal model that was developed at the University of California at Davis [23, 24], in as much as it involved crossing homozygous lean ZDF rats with obese Crl:CD (SD) rats. Although direct comparisons have not been made, the model and the disease are similar to the ZDSD and thus it is probable that it will perform similarly in many experiments. The significant advantage of the ZDSD model is that it is commercially available.

The ZDF rat is the most commonly used leptin deficient rat model of metabolic syndrome, obesity, and diabetes. Obesity is severe and is apparent very early in life. Hyperglycemia with hyperinsulinemia and dyslipidemia are apparent as early as 8 weeks of age, before the animal reaches maturity, and are sustained throughout life [25, 26]. The time course for progression to beta cell failure is rapid in this model, leading to the absence of the extended metabolic syndrome (prediabetic) condition common to human T2D. Hypertension is a major contributor to the end-organ damage seen with long standing diabetes and as such is included in the cluster related to metabolic syndrome. Reports detailing blood pressure in ZDF rats vary greatly; when assessed by radio-telemetry, blood pressure is normal [27], but hypertension has been reported when pressure is assessed by tail cuff [28]. Although marked renal damage with albuminuria is also apparent with long standing diabetes [28], this model also has significant hydronephrosis which compromises its value for application. While this rat exhibits some components of metabolic syndrome, the rats progress rapidly to overt diabetes. The largest divergence from the human T2D for this model resides in the lack of a significant prediabetic period and the development of overt diabetes in the adolescent state.

In contrast to the ZDF male, the male ZDSD rat maintains a moderately long period (approximately eight weeks) of prediabetic metabolic syndrome with obesity, followed by progression to overt diabetes and its complications. This pattern is very similar to the development of T2D in humans. The female ZDSD rat does not become diabetic on ordinary rat chow such as 5008, but under the right conditions, feeding the females diabetogenic diet (5SCA or RD12468) can trip them into overt diabetes [29].

When compared to the rodent models extensively used over the past two decades, the ZDSD rat appears to be a better translational model of obesity, metabolic syndrome, and diabetes as well as its secondary complications associated with T2D. The ZDSD rat's polygenic phenotype, as it relates to metabolic syndrome, includes visceral obesity, insulin resistance, hyperglycemia, dyslipidemia, and mild hypertension [30]. In addition, ZDSD rats spontaneously develop diabetes independently of special diets or monogenic mutations making this a much more translational model. Moreover, the development of diabetes can be synchronized with the feeding of diabetogenic diets (Purina 5SCA or Research Diets 12468) beginning at 16 weeks of age; this has been described in previously published papers [29, 31–33]. As demonstrated here, the ZDSD males become spontaneously hyperglycemic when maintained on Purina 5008 and express a heterogeneous onset of overt diabetes which mimics that seen in T2D humans. When either spontaneous or synchronized diabetes is inadequately treated, this model develops the translational complications of a T2D profile with its sequelae. The resultant naturally occurring phenotype, in the presence of a functional leptin pathway, translates to a rodent model more characteristic of the disease conditions as expressed in the human population.

The characteristics of the ZDSD rat, as it goes through the prediabetic condition (metabolic syndrome) into overt diabetes, are described in this paper. From 7 to 19 weeks of age the ZDSD male rat demonstrated obesity, impaired glucose tolerance, dyslipidemia, and prediabetic hypertension [30]. Time dependent elevation in HbA1c, which occurs during the metabolic syndrome/prediabetic phase, was also noted. Although the morning fed glucose levels are quite stable in animals 7–15 weeks of age, they are higher than those in control rats; this study did not include blood glucose from control animals. More recent data using continuous blood glucose monitoring and StatStrip reading in the am and pm have reliably demonstrated that afternoon glucose levels are significantly higher than the morning levels in this model [11]. This is most likely a representation of the abnormal feeding pattern of ZDSD rats. They are not strictly nocturnal feeders and tend to eat a significant amount during the day [34]. It should also be noted that the late afternoon glucose elevations might be related to the human dawn effect since late afternoon just before lights go off is equivalent to the rat's morning since they are nocturnal animals.

As metabolic syndrome developed in the ZDSD rat, insulin levels increased in a compensatory fashion to combat increasing insulin resistance. Shortly after the peak in insulin levels at 19 weeks of age, insulin values fell precipitously as beta cells began to fail. Progressive decreases in beta cell secretory capacity were demonstrated by the dramatic decrease in insulin and in HOMA-*β* calculated during this time. As the ZDSD model became more diabetic, further abnormalities in glucose disposal became apparent as the magnitude and duration of the glucose excursion following glucose load are increased with age, while the magnitude of the insulin response is minimized. Area under the glucose curve in the OGTT increased while area under the insulin curve was drastically diminished. In fact, in 31-week-old animals, the insulin response to glucose load was barely visible. The pattern of changes in glucose homeostasis in ZDSD is very much like that in humans as the disease progressed. Initially, obesity leads to insulin resistance due to many factors, including contributions from adipokines and cytokines from visceral fat. Glucose levels are temporarily maintained at normal level through hypersecretion of insulin from beta cells. Insulin resistance continues to progress until beta cells begin to cease functioning, insulin levels drop, and overt diabetes ensues.

Metabolic dysregulation in patients with metabolic syndrome includes hypertriglyceridemia. Similar to patients, progressive elevations in triglyceride levels were apparent in ZDSD rats. Cholesterol levels increased as animals age; interestingly, the rate of increase changed upward as ZDSD rats became overtly diabetic. Furthermore, although this study does not demonstrate the long-term complications of diabetes, we believe that this model will also develop complications that are seen in the human population with long-lasting, inadequately controlled diabetes. Indeed, in other studies, the ZDSD rat has been shown to develop diabetic osteoporosis [33], diabetic nephropathy [35], diabetic connective tissue changes [29, 32], and diabetic neuropathy [36] and hypertension prior to development of diabetes [30].

**Figure 11:**
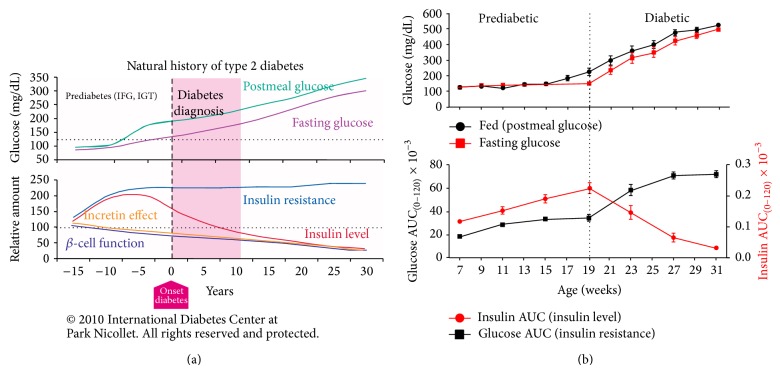
(a) The upper panel shows the graphic representation of the progression of both fasting and postmeal hyperglycemia. The lower panel demonstrates both insulin resistance and defective beta-cell function (Natural History of Type 2 Diabetes © 2010, International Diabetes Center, Minneapolis, MN, USA, used with permission). (b) demonstrates the similarity between the progression of diabetes between the human condition and ZDSD rat. The vertical dotted line represents the onset of diabetes.

A review of the literature on the development of T2D in the human population demonstrated a high degree of translation with the ZDSD model. The review and analysis by many authors have led them to an understanding of the time course of events that occur in humans in the prediabetic and diabetic conditions [15, 37–39].  Figure 11(a) captures this in a single graphic that represents the progression of the “natural history of type 2 diabetes” in the human population. This figure produced by Kendall and Bergenstal represents the changes that occur during the prediabetic stages through the overt diabetic stages of diabetes. Similar to Figures 11(a) and 11(b) graphs from this paper have combined to compare the pattern which is observed in ZDSD rats where insulin resistance is represented by the progressive decrease in glucose disposal (increase glucose AUC) and insulin response is represented by the insulin response to a glucose load during an OGTT. Insulin AUC in the prediabetic state is exaggerated while a steady decline in insulin response is noted during the diabetic state.

The current study demonstrates similarities in fasting and fed glucose levels (Figure 2), insulin levels (Figure 5), the insulin AUC (Figure 6), and the HOMA-*β* (Figure 7) in the progression of the disease from the prediabetic state to the overt diabetic state. This comparison appears to support the case for translatability of the progression of T2D in ZDSD model to the human disease.

## 5. Conclusions

The ZDSD rat model exhibits all stages and multiple components of metabolic syndrome/T2D and the complications of these conditions. Although further research is clearly needed, this study demonstrates that the ZDSD model may represent a truly translational model for the investigation of causative mechanisms, targeted interventions, and treatments for this multifaceted disease. The expression of diabetic complications in ZDSD rats may strengthen the model's translatability. The authors believe that, in addition to the scientific merits of the ZDSD, the use of the ZDSD in the “one rat, many models” paradigm presents an opportunity for investigators to significantly reduce costs while evaluating compound effects on the multiple components of T2D and its sequelae.

## Figures and Tables

**Table 1:** Profile of spontaneous hyperglycemia in individual ZDSD rats (6:00 a.m. fed BG levels, mg/dL).

Animal ID	7 weeks	9 weeks	11 weeks	13 weeks	15 weeks	17 weeks	19 weeks	21 weeks	23 weeks	25 weeks	27 weeks	29 weeks	31 weeks
1	121	**130**	**133**	**149**	**140**	112	**163**	**230**	*270 *	*375 *	*472 *	*487 *	*527 *
2	111	**130**	120	**133**	124	**129**	113	**129**	**172**	**234**	*407 *	*487 *	*505 *
3	**139**	**146**	**128**	**158**	123	**138**	**136**	**237**	*296 *	*341 *	*406 *	*443 *	*494 *
4	**133**	**125**	124	**137**	**133**	**160**	**133**	*313 *	*367 *	*405 *	*482 *	*537 *	*531 *
6	**134**	**127**	116	**151**	**144**	**140**	**140**	**172**	**180**	*324 *	*456 *	*463 *	*483 *
7	**140**	**131**	**133**	**146**	**158**	**197**	**153**	*438 *	*472 *	*502 *	*535 *	*523 *	*531 *
8	114	**131**	106	**131**	**125**	**147**	**198**	*301 *	*378 *	*408 *	*489 *	*495 *	*545 *
9	122	**144**	119	**152**	**160**	*292 *	*395 *	*394 *	*514 *	*541 *	*535 *	*593 *	*564 *
10	**131**	**154**	120	**162**	**169**	*265 *	*322 *	*404 *	*490 *	*451 *	*496 *	*592 *	*524 *
11	**133**	123	**140**	**168**	*326 *	*405 *	*442 *	*511 *	*555 *	*544 *	*545 *	*536 *	*592 *
12	112	123	118	**141**	**147**	**207**	**247**	*411 *	*446 *	*461 *	*501 *	*566 *	*621 *
13	**129**	**138**	**134**	**146**	**148**	*265 *	*366 *	*453 *	*438 *	*507 *	*600 *	*502 *	*572 *
14	**141**	**143**	**128**	**157**	**171**	**199**	*325 *	*386 *	*447 *	*493 *	*509 *	*540 *	*514 *
15	**133**	**126**	**128**	**134**	**141**	*278 *	*365 *	*436 *	*507 *	*544 *	*578 *	*526 *	*584 *
16	114	**135**	**129**	**148**	**148**	**178**	**142**	97	**210**	*322 *	*473 *	*427 *	*465 *
17	118	117	108	**138**	**135**	**149**	**132**	**169**	**144**	*371 *	*472 *	*457 *	*483 *
18	**140**	123	116	**133**	**153**	**176**	**219**	**140**	**235**	121	*393 *	*391 *	*515 *
19	**129**	**131**	115	**141**	**139**	**158**	*287 *	*363 *	*423 *	*451 *	*525 *	*511 *	*549 *
20	107	**142**	**126**	**188**	**162**	107	*284 *	*379 *	*416 *	*463 *	*487 *	*541 *	*531 *
21	124	**141**	112	**143**	108	114	**138**	**189**	*410 *	*424 *	*458 *	*472 *	*511 *
22	**136**	**129**	**140**	**133**	116	120	**128**	**225**	**231**	*295 *	*427 *	*454 *	*457 *
23	123	**129**	**125**	**138**	**159**	**132**	113	124	**221**	**180**	**233**	*303 *	*407 *
24	**146**	**131**	116	**140**	118	**207**	*286 *	*397 *	*508 *	*494 *	*525 *	*575 *	*635 *

Values are represented as different fonts as each animal becomes progressively hyperglycemic. Normal represents relatively normoglycemic, bold represents hyperglycemic (125–249 mg/dL), and italic represents diabetic and overtly diabetic (>250 mg/dL).
